# Molecular T-Cell Repertoire Analysis as Source of Prognostic and Predictive Biomarkers for Checkpoint Blockade Immunotherapy

**DOI:** 10.3390/ijms21072378

**Published:** 2020-03-30

**Authors:** Ilenia Aversa, Donatella Malanga, Giuseppe Fiume, Camillo Palmieri

**Affiliations:** 1 Research Center of Biochemistry and Advanced Molecular Biology, Department of Experimental and Clinical Medicine, University “Magna Græcia” of Catanzaro, 88100 Catanzaro, Italy; ilenia.aversa@unicz.it; 2 Interdepartmental Center of Services (CIS), Department of Experimental and Clinical Medicine, University “Magna Græcia” of Catanzaro, 88100 Catanzaro, Italy; malanga@unicz.it; 3 Department of Experimental and Clinical Medicine, University “Magna Græcia” of Catanzaro, 88100 Catanzaro, Italy; fiume@unicz.it

**Keywords:** T-cell repertoire, checkpoint blockade immunotherapy, TCR clonotype

## Abstract

The T cells are key players of the response to checkpoint blockade immunotherapy (CBI) and monitoring the strength and specificity of antitumor T-cell reactivity remains a crucial but elusive component of precision immunotherapy. The entire assembly of T-cell receptor (TCR) sequences accounts for antigen specificity and strength of the T-cell immune response. The TCR repertoire hence represents a “footprint” of the conditions faced by T cells that dynamically evolves according to the challenges that arise for the immune system, such as tumor neo-antigenic load. Hence, TCR repertoire analysis is becoming increasingly important to comprehensively understand the nature of a successful antitumor T-cell response, and to improve the success and safety of current CBI.

## 1. Introduction

Checkpoints blockade immunotherapy (CBI) have demonstrated unprecedented therapeutic benefits in a significant percentage of cancer patients [1,2]. CBI uses antibodies that block inhibitory immune pathways that protects at least some cancers by helping to establish and maintain the immunosuppressive microenvironment [3]. Despite demonstrated success, CBI shows major limitation, including low response rate and drug resistance, and a major remaining challenge is identifying which patients will respond to CBI and defining the reasons for success versus failure of the treatment [4]. Therefore, in clinical practice has arisen the need for biomarkers and methods that guide patient selection, to provide early treatments indicators of response, and to predict therapeutic effects and adverse events [3,5].

The T cells are key players of the response to CBI [3], and monitoring the strength and specificity of antitumor T-cell reactivity remains a crucial but elusive component of precision immunotherapy. The main molecular determinant of T cells, ensuring antigen specificity and strength of the immune response, is the variable region of the T-cell receptor (TCR), the result of inherently stochastic genetic recombination and diversification mechanisms occurring during T-cell development in thymus. Starting from a large collection of gene segments, the rearrangement process ensures that each mature and functional T cell is endowed with a unique TCR sequence. As a T cell is activated in periphery and undergoes a clonal expansion, all the cells of the clonal lineage are equipped with an identical TCR sequence.

The entire assembly of TCR sequences in an individual, that is the combination of their identities and distribution, is referred to as the TCR repertoire, the scale of which is enormous, estimated to be in the range of about 10^14^-10^19^ different TCRs [6].

The TCR repertoire hence represents a “footprint” of the conditions faced by T cells that dynamically evolves according to the challenges that arise for the immune system, such as tumor neo-antigenic load. Moreover, TCR repertoire represents a source of potential high-dimensional biomarkers for tumor development and personalized predictor of the efficacy of immunotherapies [7]. Hence, TCR repertoire analysis is becoming increasingly important to comprehensively understand the nature of a successful antitumor T-cell response, and to improve the success and safety of current CBI.

Assessing the TCR repertoire requires profiling highly heterogeneous T cell populations at the single-cell level in a high-throughput manner, something that until recently was unthinkable to pursue due to the strong technological limitations. The incredible advancement of high-throughput sequencing (HTS) technologies now allows us to analyze the TCR repertoire at a deeper and finer level than traditional assays such as flow cytometry and spectratyping [8]. By sequencing the variable determinants of TCR, which represent an accurate identifier of the majority of T cells, these technologies can provide the full representation of the TCR repertoire in a sample.

The exponential rise in TCR repertoire data has also catalyzed the field of computational and systems immunology, leading to a large increase in the number of computational methods directed at dissecting repertoire complexity [8]. Sequencing data can be used to assess the clonal richness and diversity of lymphocyte populations; to track specific clonotypes over time, between tissues, and across lymphocyte subsets; to detect clonal expansion; and to detect the recruitment of new clones into a tissue. This review provides the general concept of CBI (Section 2) and T-cell immune repertoire (Section 3); summarizes the methodologies and bioinformatics tools for TCR repertoire analysis (Section 4 and Section 5); and describes the current state of knowledge about the biological and clinical significance of the TCR repertoire in the context of the CBI (Section 6).

## 2. Checkpoint Blockade Immunotherapy

**Figure 1:**
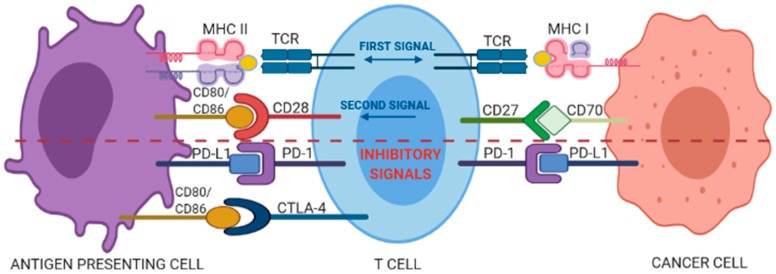
Interplay between co-stimulatory and inhibitory signals of a T-cell interacting with an antigen presenting cell (APC) or a cancer cell. See text for details.

T cells can identify and destroy nascent tumor cells by recognizing tumor-specific antigens presented by the major histocompatibility complex (MHC), a process known as cancer immunosurveillance [9]; in fact, evasion of immunosurveillance is considered a peculiar hallmark of cancer [10]. The amplitude and quality of the T-cell response is regulated by a fine balance between co-stimulatory and inhibitory signals, the last known as immune checkpoints [11]. The first co-stimulatory signal confers specificity to the immune response, being accounted by the recognition of antigenic peptide/HLA complexes on the surface of antigen presenting cells (APCs) by the antigen-specific TCR. The second type of signal is supplied by the so called “immune checkpoint” molecules, which dynamically regulate the magnitude of the response through co-stimulatory or inhibitory signals (Figure 1). 

The CD28 co-stimulatory receptor, constitutively expressed in 80% of the CD4^+^ T cells and in 50% of the cytotoxic CD8^+^ T cells, binds to cognate CD80 and CD86 ligands (also called B7-1 and B7-2, respectively) expressed by APCs. This binding starts intracellular signals that activate transcriptional factors such as NF-AT, AP-1, and NF-κB, which in turn determine the clonal expansion of activated T cells and their differentiation into effector and memory T cells. In contrast, the cytotoxic T lymphocyte-associated antigen 4 (CTLA-4), a CD28 homologue with 31% sequence identity, delivers inhibitory signals for down-regulation of immune responses [12] and binds with much higher affinity to CD80/86 ligands as compared to CD28, thus competing the CD28-mediated co-stimulatory signal. Furthermore, it is able to wipe out the two ligands via trans-endocytosis limiting CD28-mediated activating signalling [13]. Another important antigen-independent co-inhibitory receptor is the Programmed Death-1 (PD-1, also known as cluster of differentiation 279 (CD279)) [11]. The binding of PD-1 with either one of its two known ligands, programmed death-ligands 1 and 2 (PD-Ll or PD-L2), induces inhibitory signals reducing T-cell activity and proliferation, modulating the production of IFN-γ, TNF-α, and IL-2, and increasing T-regulatory cells suppressive activity [14,15]. Beside these “first-generation” immune checkpoint pathways, an array of additional inhibitory or co-stimulatory molecules populate the tumor microenvironment and are currently being investigated as targets for new immune checkpoint blockade drugs [16].

Hence, the immune checkpoint molecules provide the optimal balance between immune responses to antigens and maintenance of self-tolerance under normal physiological conditions. In pathological conditions, the cascade of molecular signals operated by immune checkpoint molecules, are hijacked by viruses, or tumor cells, to suppress the immunosurveillance. The immunological tolerance that establishes within tumor microenvironment, driving the development of tumor towards the clinically evident phases, brings significant benefits to the mechanisms that maintain the activation of the inhibitory immune checkpoint pathways [11].

Since the discovery of immune checkpoint pathways in the last decade of the twentieth century, and the knowledge of their role as a hinder to the ability of the immune system to fight cancer, it was evident that blocking this negative regulation might restore immune surveillance and enhance the immune system to attack tumors efficiently. In a seminal paper in 1996, Leach et al. showed for the first time that antibodies against CTLA4 could help reject pre-established tumors in mice [17]. Since then, immune checkpoints inhibitors have been considered as novel targets for cancer immunotherapy, and several antibodies targeting CTLA4, PD-1, and PD-L1 are currently approved for use in a variety of different cancers (reviewed in [18,19]). Several clinical trials have demonstrated that CBI improves the overall survival and long-term safety in a number of different cancers, as well provoking fewer metastases and adverse effects as compared with traditional cancer treatments such as chemotherapy and radiation therapy [1,20,21,22,23,24]. Multiple additional trials using CBI drugs either as single agents or in combination with other agents are ongoing to extend their indication areas [18,19].

In choosing CBI over “conventional” treatments, the fundamental parameters for success of therapy such as mutational load, the presence of lymphocytes within the tumor and the expression levels of immune checkpoint molecules (e.g., PD-L1,) must always be taken into consideration [25,26,27]. Some limitation still have to be overcome in order to become the treatment of choice in the some malignancies, such as the costs of production, conservation, stability, and immunogenicity [28]. Perhaps the biggest challenge among them is to pass the concept of resistance [29]. The resistance develops in 50% of the patients treated with immunotherapy and consists of a very dynamic and complex mechanism, such as the constitutive expression of PD-L1 on cancer cells, new tumor antigens presentation, epigenetic modifications and modulation of the tumor microenvironment toward a tolerogenic status [30,31]. Hence, a major remaining challenge is identifying which patients will respond to CBI and defining the reasons for success versus failure of the treatment, contexts in which the analysis of the TCR repertoire may provide a promising contribution.

## 3. The Immune Repertoire

The immune repertoire is a concept that peculiarly pertains to the acquired immune system, and is grounded on the capability of T and B cells to potentially recognize any structural determinant of an antigen. This possibly infinite potential pattern of recognition is accounted by mature and functional cell surface TCR and B-cell receptor, which result from inherently stochastic combinatorial genomic recombination and additional random diversification mechanisms. Hereafter, we focus only on the TCR, the main subject of this review.

**Figure 2:**
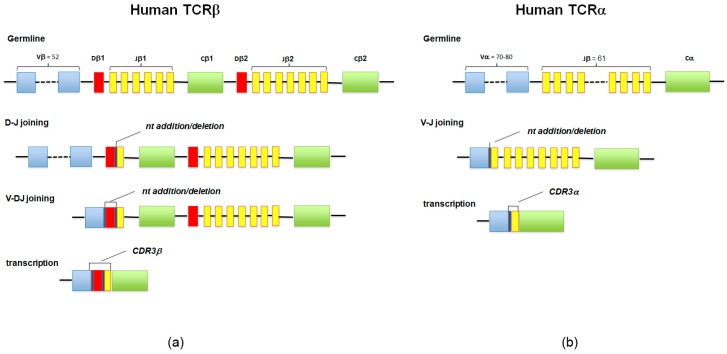
(**a**,**b**) The diversity of T-cell receptor (TCR)αβ is a result of genetic recombination and diversification mechanisms occurring at the α and β TCR chain loci. Diversity is first created in the germline via recombination of variable V, diversity D (for β chain), and joining J segments. Further diversification occurs through imprecise junctions of these gene segments (addition of P- and N-nucleotides adjacent to the D segment), and the combination of α and β chains.

T cells express a heterodimeric TCR consisting of an alpha (α) chain and a beta (β) chain (TCRα/β, about 95% of peripheral T cells), or gamma (γ) and delta (δ) chains (TCRγ/δ, about 5% of peripheral T cells). The TCR chain loci are organized in a set of gene segment families: variable (V) and joining (J) gene segments for α and γ chains, and additional D gene segments for β and δ chains. The β and δ chains are assembled by means of the VDJ recombination that randomly combines V, D, and J, whereas for α and γ chains only VJ recombination occurs (Figure 2).

During this process, the junctions between these segments are often modified by stochastic deletions and insertions of random, untemplate nucleotides, a process that confers further diversity. This highly diverse junctional region of the TCR chain, also known as the complementarity-determining region 3 (CDR3), is an important determinant of antigen recognition [32]. The aspect more strictly concerning the subject of this review is that the CDR3 sequence is essentially unique for each newly formed T cell, since it is highly unlikely that two T cells will express the same CDR3 nucleotide sequence [33]. At the same time, when a T cell is activated and undergoes a clonal expansion, all the cells of the clonal lineage are equipped with an identical CDR3, which therefore acts as a natural identifier of the clonality of the lymphocytes [34]; the term “clonotype” refers to the characteristic TCR sequence that identifies a T cell clone.

The human TCR repertoire refers to the whole range of different TCRs present in an organism. The genetic diversification mechanisms acting on a small set of genes that encode the TCR (recombination, random insertion, and deletion) have the potential to create 10^14^-10^19^ new genetic configuration of TCRα/β, according to probabilistic models of TCR rearrangement, where diversity is largely dominated by the CDR3 [6,35].

Despite the high theoretical diversification potential of the TCR, approximately 4×10^11^ T cells circulate in the adult human body [36], with an estimated number of clonotypes ten times lower (about 10^10^) [37]. Various cellular selection mechanisms (e.g., thymic education or antigen specificity) reduce the actual diversity of the TCR repertoire, including the not completely biased V-region usage during V(D)J recombination and antigen selection [35].

The need for accurate methods for individual TCR repertoire is justified by the prevalent representativeness of rare clones, and a minimal presence of TCRs that are common in the general population (“public” clonotypes). Thus, for a reliable representation of TCR repertoire, analytical methodologies should ensure an analytical accuracy that exceeds the molecular diversity, or at least, the clonal diversity of the underlying sample [38].

## 4. Methodologies for TCR Repertoire Analysis

For many years the lack of reliable and robust high-throughput technologies has limited the study of human TCR repertoires. In recent years, the incredible advancement of highly specific HTS methods has facilitated the parallel analysis of millions of TCR sequences. Several sequencing platforms now offers kits and service for TCR repertoire analysis (Table 1).

**Figure 3:**
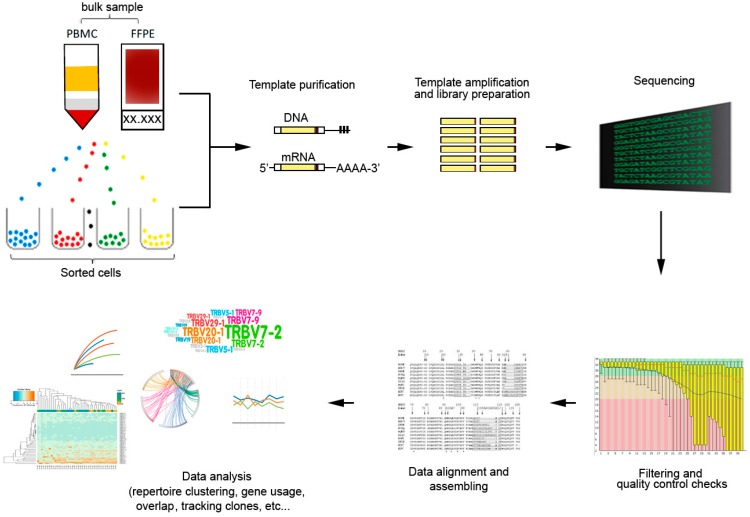
General workflow for TCR repertoire sequencing and analysis. From bulk samples (tissues or peripheral blood) or sorted cells, genomic DNA of mRNA templates are isolated and amplified by polymerase chain reaction (PCR) with specific primers to generate to generate the TCR library. High-throughput sequencing generate the TCR sequencing data that can be analyzed with bioinformatics tools based on different research objectives.

In its simplest form, they analysis comprise of three essential working steps: (i) PCR amplification of V-D-J (for TCRβ) or V-J (for TCRα,) gene segments; (ii) massively parallel sequencing of the PCR amplicons; and (iii) alignment of HTS reads by bioinformatics tools (Figure 3).

However, at present it is still difficult to define a gold standard method, like all available the method has its advantages and disadvantages. A detailed description of all the HTS methods available today, their advantages and disadvantages, goes beyond the real topic of this review. In this regard, we refer to recent and excellent reviews [39].

Several pitfalls in the experimental workflow can affect the biological conclusion of a study, among which biological sampling and technological sampling have a more relevant impact [38]. In addition, limited sampling from peripheral blood or particular tissues/organs always raises the problem of “unseen clones.” Furthermore, bulk methods are not able to exactly match the information on the variable region that determine the specificity of the antigen of each clonotypes, a limitation that can be overcome by the more expensive and laborious single cell approaches [40].

The biological sampling issue refers to the fact that repertoire diversity is substantially dependent on the comprehensive sampling of the cell population studied, and that insufficient biological sampling results in capturing only a portion of the TCR repertoire [41]. To limit the biological under-sampling it is important that the cell population sampled must be an approximate representation of the cellular compartment being investigated, which could be realized by using different samples of the same underlying cell population (biological replicates) [41]. Also, real-world experiments will always exhibit some degree of experimental error; indeed, the possibility of sequencing errors and amplification bias is theoretically unavoidable because it is inherent in PCR-based HTS platforms and methods for library preparation [38]. Other sources of error include annotation error introduced during data processing (see below). So, measuring diversity accurately requires methods that address the experimental noise as well. As a general indication, the experimental plan should ensure that the number of sequencing reads exceeds the molecular diversity, or at least, the clonal diversity of the underlying sample. To achieve this goal, it is important to perform replicate sequencing of the same immune repertoire library, that is resequencing of the same library, sequencing separate library preparation of the same genetic material [42]. Thus, the diversity observed in an experimental sample (e.g., a tumor biopsy, a peripheral blood drawn) does not reflect the actual diversity realized in the whole organism, due to rare clonotypes, sequencing sensitivity, and accuracy, so that the biological conclusiveness of diversity and clonality analysis will benefit from the implementation of biological and technological replicates.

The preferential target of many TCR repertoire studies is the CDR3, due to the high diversity of the V(D)J recombination junctional site and its relevance for TCR peptide interaction. However, the sequence of the entire TCR transcript, thus including CDR1 and CDR2, may be a great advantage for modelling the TCR structure and its binding properties. In fact, even though CDR1 and CDR2 do not directly interact with the antigen, they play an important role in making contact with the MHC molecule and thus influence the sensitivity and affinity of the TCR binding [43,44]. Methods based multiplex-PCR, which make use of many allele-specific primers annealing in different positions of the V genes, are almost unable to also detect CDR1 and CDR2. This limitation can be overcome by methods based on rapid amplification of 5′ complementary DNA ends (5′RACE) cDNA synthesis and nested PCR. These approach allows the synthesis of cDNA strands containing the complete 5′ end of the mRNA, independent of the carried V allele, thus enabling the capture of all TCR variants that include CDR1 and CDR2. The following PCR step may be carried out using a common adaptor as 5′ primer and constant region primers for the 3′ end. The PCR products can be ligated to the appropriate sequencing adaptors and used for HTS sequencing.

It is important to mention that every method based on PCR are subjected to amplification biases, due to a preferential amplification of some alleles compared to others [45]. Moreover, the sequencing process has an intrinsic error rate that is independent of the library preparation, but dependent on sequencing depth of the choice platform. Since a specific TCR may differ from another by only a single nucleotide, it is very challenging to distinguish among PCR errors, sequencing errors and low frequency clonotypes [46,47]. So far, the main approach used to overcome these issues is the usage of unique molecular identifiers (UMIs), introduced into each cDNA molecule, that enables the accurate computational correction for sequencing and PCR errors and bias [48,49,50,51]. Specific algorithms are applied for correction of this particular type of data [52].

Raw sequencing reads from HTS machine are first pre-processed for quality reads and filtering (e.g., barcode demultiplexing and adapter trimming), and then subjected to alignment with a reference database for annotation, the most important being the IMGT (International Immunogenetics Information System), which are able to provide comprehensive information (e.g., germline gene usage, framework regions, CDRs) [53]. So far, many bioinformatics framework are available for aligning TCRα/β sequences and quantifying for downstream statistical analysis (Table 2) [54].

## 5. Analysis of Immune Repertoire

Major goals of the TCR repertoire analysis is the quantification of such a changes in repertoire diversity, yielding information on the current status of an immune response. Data list of annotated clonotypes are processed by additional software tools (e.g., immunarch [62], immunoMap [63], VDJtools [64], and R LymphoSeq [65]), which will evaluate the characteristics of the various individual repertoires, including diversity of repertoire, clonal size distribution, and use of specific gene segments (see below). Through this analysis, the complexity of the sequencing data is commonly summarized in metrics of repertoire diversity and clonality that, in the context of a clinical study, can easily be related to (i) prognostic factors; (ii) evolution of disease; and (iii) response to therapies. Noteworthy, the mathematical foundations and terminology used for immune repertoire analysis derive from ecology, where they are used to quantify species diversity and composition [66].

The diversity measurement relates to the number of species (clonotypes) present in a biological entity. The simplest and most familiar diversity measure is the species richness, which reflects the total number of species (for example, V-J rearrangements, CDR3 amino acid or nucleotide sequences). However, species richness is that measure which more than others is affected by the biological under sampling and technological issues. A strategy to avoid the impact of sequencing depth differences on the quality of richness estimation, is to sort the reads by clone abundance and calculate the number of unique clonotypes comprising the top part of cumulative reads (e.g., the top 25% percentile). This metric is not strongly influenced by rare clonotypes, thus is amenable to comparisons between experiments [41].

Another caveat of species richness is that it does not take into account the relative frequency of each clonotypes, thus resulting in an inadequate description of the repertoire diversity; in fact, two populations can have the same number of clonotypes (species richness), but each of them can be present with a different frequency in the TCR repertoire. T-cell clonal space is defined as the summed frequency of clones in the total TCR repertoire. T cell clones that have expanded following their encounter with the cognate antigens will produce a bias in the clone size distribution, where a relatively small number of clones will be more represented within the clonal space. Summarizing, the population diversity of the TCR repertoire can be quantitatively expressed by two separate factors: richness (i.e., the number of unique elements in a population) and evenness (i.e., the frequency distribution of those elements).

In order to measure diversity in a more complex way than using the simple number of species, some diversity indices have been introduced that take into account both richness and evenness. These indices are all related to the Rényi entropy, a family of diversity measures initially developed for information theory, which quantifies the uncertainty in predicting the sequence identity of a random sequence from a dataset [67]. When Rényi entropy function is applied to clonotype frequencies, it gives rise to a number of parameterized indices, each capturing different part of the distribution of clonotype frequencies data, i.e., some of them rely very strongly on correctly capturing the tail of rare clonotypes, while other measures systematically down-weight or undercount rarer clones [67]. Therefore, these indices provide complementary information on the size-frequency distribution of clonotypes in the population, and they can be selectively used depending on the biological demand being addressed. For example, the Shannon diversity index (Shannon’s entropy) is defined as:

$$H=-\sum_{i=1}^{N}pi\ln pi$$

where *pi* is the proportion of sequence *i* relative to the total N sequences. It accounts for both richness of the sample (i.e., the number of unique TCR/CDR3 sequences) and relative abundance (evenness) [68,69]. A large Shannon diversity index reflects a more diverse distribution of the CDR3 sequences.

The comparison between different samples using the Shannon diversity index must be used with caution, because it assumes that the distributions of the clonal frequencies of the samples are similar to each other. The Pielou’s evenness index represents a normalization of the Shannon diversity index by division of log 2 of the number of unique productive sequences:

J=H/log(S)

where *H* is the Shannon index and S is the number of unique TCR/CDR3 sequences. The Pielou’s evenness index thus allows for comparisons between samples differing in the total number of reads: high evenness (on a scale of 0 to 1) implies an almost uniform distribution, whereas low evenness is indicative of population skewing due to the biased expansion of individual T cell clonotypes.

The Inverse Simpson index is the effective number of types that is obtained when the weighted arithmetic mean is used to quantify average proportional abundance of types in the dataset of interest: High values indicate even distribution of TCR clones, and low values indicate enrichment of T cell clones.

Repertoire diversity can be also assessed using clonality scores, the simplest one being the clonal proportion, that is the fractional (percentage) composition of an individual clonotype relative to the total number of clonotypes. Most commonly, clonality scores are derived from Shannon’s entropy clonality can be calculated from entropy of the clonotypes frequency distribution (Shannon’s entropy), and the normalized by the log of richness. The inverted metric (1—normalized entropy) result in a clonality value that ranges from 0 (the most diverse repertoire, or polyclonal repertoire, that is every T-cell in a sample contains a unique TCR), to 1 (monoclonal distribution) [68,69]. Anyway, the meaning of clonality statistics are just the inverse of the diversity statistics, such that higher clonality typically means lower diversity.

Gini coefficient, commonly used as a measure of income inequality in economics, can be used to assess the inequality of clonotype distribution within a repertoire [70] (Table 3).

Repertoire overlap is the most common approach to measure repertoire similarity. It is achieved by computation of specific statistics on clonotypes shared between given repertoires, also called “public” clonotypes. The repertoire overlap can be used to measure the change between sequential experiments. A therapeutic effect on the TCR repertoire would result in a lower degree of repertoires overlap before and after therapy, as compared to the higher overlap expected for repertoires not impacted by the therapy. The Morisita’s distance to clone count distributions is often used to quantity the overlap between two populations; precisely, Morisita’s distance is an inverse measure of overlap so that two population with the greatest overlap will have a minimal Morisita’s distance, while two very different repertoires will show the maximum Morisita’s distance [38]. This index has been frequently used to quantify the repertoire change between sequential experiments, e.g., the repertoire of a patient before and after treatment [72].

The uniqueness of the CDR3 TCR sequences enable tracking of clonal expansion or contraction in serially sampled tumors. Clonotype tracking is popular approach to monitor changes in frequency of clonotypes of interest. For example, a specific clonotype can be tracked across different time points in pre- and post-treatment repertoires. The most frequent and feasible way for tracking clones in peripheral blood is to focus on the most abundant clonotypes (e.g., 25%) [71,85], thus avoiding the noise from the most infrequent clones. Another strategy for TCR clonotype dynamics during CBI is to focus on peripheral TCR clones that show a differential abundance between baseline and the defined time point (e.g., after each dose of drug, or objective clinical responses) [81]. The analysis can be refined by considering the peripheral TCR clones also found in the tumor [86]. The average productive frequency of these differentially abundant clones can be used as a metric of TCR dynamics during therapy [86]. The main TCR repertoire metrics used as biomarkers in major CBI studies are summarized in Table 3.

A further level of analysis of the repertoire regards the search TCR sequences with specific antigenicity known, or the predictive analysis of TCR antigen specificity according to their sequence [87,88]. The prediction of the TCR antigen specificity based on the TCR amino-acid sequence alone is extremely challenging. This issue has been recently addressed by generating a large database of antigen-specific TCR sequences and elaborating algorithms that accurately identify the patterns of sequence motifs that correlated with antigen specificity [89]. By this approach, it has been possible to identify neoantigen-specific T-cell clones in the neoadjuvant setting and to track neoantigen-specific T-cell clones in blood upon anti-PD-1 therapy [75]. In this regard, it is important to emphasize that most of these studies are based on an approach that focuses attention on the T-cell response towards tumor neoantigens arising from non-synonymous mutations. In this regard, the analysis of the TCR repertoire can allow the quantification of convergent TCRs, that is TCRs endowed with a shared CDR3, and thus antigen specificity, but different nucleotide sequences [84]. The phenomenon of TCR convergence arises from chronic antigen stimulation that results is a spectrum of T cells with different TCR sequences but the same antigen specificity; thus, quantification of convergent has been used to infer the presence of tumor antigen specific T-cell, beyond those directed against non-synonymous mutations [84]. TCR convergence can be calculated as the aggregate frequency of clone with unique TCRβ nucleotide sequences sharing a variable gene and CDR3 amino acid sequence with at least one other identified clone.

Finally, it is important to mention that the diversity of TCR repertoire can be analyzed by considering either the VJ cassette combination, or the amino acid CDR3 sequences encoded by each VJ cassette combination. These two possibilities should not be considered redundant because they express two distinct components of the total diversity of the repertoire: (i) VJ-dependent component, which is strongly influenced by developmental and lineage restriction, and (ii) VJ-independent component, which includes selection for the antigen-binding affinity of the CDR3 sequence [90].

## 6. The Impact of CBI on TCR Repertoire

### 6.1. Metastatic Melanoma

The first reports on TCR repertoire analysis in the settings of CBI focused on the peripheral TCR diversity and clonality in metastatic melanoma patients [71]. By using TCRβ HTS in blood of patients treated with an anti-CTLA4 antibody, Robert, L. et al. demonstrated a remarkable effect of this therapy expanding the number of TCRβ in blood. In particular, the anti-CTLA4 resulted in increased diversity indices (richness and Shannon diversity index) upon treatment, and the higher diversity indices correlated with clinically observed toxicities but not with clinical response [71]. A similar lack of association between baseline peripheral TCR repertoire diversity and response was recently observed in patients treated with combination therapy of anti-CTLA4 and interferon-α [83]. An opposite findings was obtained by Postow et al., who reported that patients who had clinical benefit had a higher degree of richness and evenness in their peripheral baseline TCR repertoires than patients who did not have clinical benefit [77]. Although these studies utilized different CTLA-4 blocking antibodies (tremelimumab in [71,83] and ipilimumab in [77]) a more plausible explanation for the discrepant results would be due to the different methodologies they used, thus underlining even more the need to optimize and validate the methodology for the analysis of the T cell repertoire.

Cha et al. focused on the 25% most abundant clonotypes in pre-treatment peripheral sample, and determined the fold changes for paired pre- and posttreatment patient samples, as well as untreated control samples, separated by the same time interval [72]. They observed that ICB treated patients showed a greater extent of fluctuation in clonotype abundance (either increase, or decrease) after one cycle of anti–CTLA-4 treatment than untreated control subjects, and that a reduced loss of clonotypes was associated with the improved clinical outcomes. These results indicate that maintaining high-frequency TCR clonotypes during treatment would have a positive impact on the clinical course of the disease [72].

Beside diversity and clonality, TCR convergence in pre-treatment peripheral has been used as a predictive biomarker for response to CTLA-4 blockade in a cohort that included melanoma patients, demonstrating a good diagnostic specify and sensitivity in discriminating responders from non-responders [84].

When focusing on intratumor TCR clonality, Roh et al. did not observed any difference when comparing responders to non-responders in the context of CTLA-4 blockade at the pre-treatment and on-treatment time points [80]. However, when they compared clonality in patient-matched sequential tumor samples, an increase in clonality was noted in a subset of patients who subsequently responded to anti-PD1 therapy [80].

A more clonal and less diverse intratumor TCR repertoire was found in responding patients treated with anti-PD-1 [73,80]. Biopsies from patients that experienced radiographic response to therapy showed an enriched population of T-cells with unique specificities [73]. When TCR clonality (1—Pielou’s eveness) was assessed at baseline and post-dosing biopsies, responder patients showed more than ten times as many clones expanded than progressors [73].

The most frequent tumor-infiltrating clonotypes were readily identifiable in the blood and after anti-PD1 CBI, regardless of clinical response [91]; some of them showed a Ki67+ (HLA-DR+CD38+) T exhausted phenotype, supporting the notion that T exhausted cells in the blood are reinvigorated by anti-PD-1 therapy and contain T-cell clones that are also present in the tumor [91].

Previous reports indicate that a high tumor mutation load may increase the probability of generating immunogenic neoantigens, which elicit effective immune responses [92,93,94]. Indeed, in melanoma patients neoantigens T cells have been identified in the circulating PD-1+/CD8+ T-cell population that matched with tumor-resident PD-1+/CD8+ T-cell [91].

A recent clinical study has made it possible to evaluate the association between the diversity of the T-cell repertoire and the response to nivolumab in two groups of patients: those who relapsed from ipilimumab (anti-CTLA4) treatment (ipi-P), and those who were treatment free (ipi-N). The analysis of TCR repertoires suggested that anti-PD-1 response is associated with different patterns of T cell diversity dynamics in Ipi-N versus Ipi-P patients. Pre-therapy diversity indices (richness and evenness) were not different between the two groups, neither among response status to nivolumab. However, the median fold change in the number of unique CDR3 sequences (richness) was significantly associated with benefit upon therapy (complete/partial response or stable disease) in ipi-P patients, but in not in ipi-N patients. In contrast, the median change in T cell evenness on-therapy was associated with benefit in Ipi-N but not Ipi-P patients.

### 6.2. Lung Cancer

The first conflicting results on the prognostic and predictive significance of peripheral TCR diversity and clonality in metastatic melanoma may be due to the existence in the whole TCR repertoire of a large number of non-tumor specific TCRs that dilute tumor neoantigen specific TCRs. Starting from this assumption, Han J. et al. thought to focus on PD-1+/CD8+ exhausted T cells [82], since this cell subset seems to include the highest number of cytotoxic T cells specific for neoantigen [95]. They found that pretreatment TCR diversity of sorted peripheral PD-1+/CD8+ T cells predicted clinical response to anti PD-1/PD-L1 CBI in non-small cell lung cancer (NSCLC), since patients with a higher diversity had a significantly longer progression-free and overall survival than those with lower diversity. Therefore, a greater diversity of exhausted T cells, which probably have a high frequency of tumor neoantigen specific T-cells, is indicative of a higher probability that these tumor neoantigen specific T-cells can be reinvigorated by the CBI, experiencing clonal expansion and leading to an improved immune response. This interpretation is supported by the dynamics of peripheral PD-1+/CD8+ TCR repertoire during treatment. In fact, the authors found that TCR clonality of sorted PD-1+/CD8+ T cells positively associated with survival outcomes, that is patients with increased clonality had improved survival outcomes as compared to those with decreased TCR clonality [82]. Overall this study point to the PD-1+/CD8+ T cells as source of predictive biomarkers with clinical utility.

An initial analysis of TCR repertoires in sorted cell from tumor and matched normal lung tissues in a cohort of 47 NSCLC patients, has allowed to elucidate the association between TCR diversity and the prognosis of lung cancer patients [96,97]. Most T cell clones had extremely low frequencies, with a similar distribution between cancer tissues and normal lung tissues; even though only a small proportion of T cell clones were highly expanded clones (frequency > 0.1%), their rates were higher in normal lung tissues than in tumor tissues. The diversity of intratumor T-cell clone, expressed as inverse Simpson’s diversity index, was higher in tumor tissues than in normal tissues, but no significant differences were observed regarding the associations of the TCR diversity with tumor stage and differentiation stage [97].

The most relevant knowledge on the biological significance and clinical utility of T-cell repertoire analysis is emerging from the study on the safety and feasibility of neoadjuvant anti-PD-1 therapy in early stage (stage I, II, or IIIA) NSCLC (NCT02259621) [75]. The neoadjuvant setting, whereby CBI blockade is given before surgical resections, has provided the unique opportunity to monitor the TCR repertoire across time (in serial peripheral blood draws) and space (across different biological compartments) according to pathologic response. The authors used matched tumor, normal lung tissue, and longitudinal peripheral blood samples to compare the quantitative and qualitative changes in the T cell repertoire of responders versus non-responder, by using the TCR as a molecular barcode. The treatment led to peripheral expansion of multiple T-cell clones that were also found in the tumor at the time of resection. Many of these clones were not detected in the peripheral blood before treatment. Then, the authors addressed the hypothesis that a substantial component of antitumor immunity after PD-1 blockade is directed toward mutation-associated neoantigens. To this end, they used a web-based bioinformatics platform that identify mutation-associated neoantigen-specific T-cell clones [89], and demonstrated that in a patient showing a complete pathological response, T-cell clones specific for mutation-associated neoantigens were rapidly expanded in peripheral blood after neoadjuvant PD-1 blockade [73].

The continuation of this study presented a more exhaustive characterization of the dynamics of the repertoire in relation to neoadjuvant PD-1 blockade [98]. Tumor clonality positively correlated with tumor mutational burden, and inversely associated with residual tumors, thus supporting the hypothesis that a high tumor mutational burden increases the likelihood that neoantigens can drive a clonally skewed intratumor T cell repertoire leading to tumor pathological regression. Then the authors showed that the top 1% most frequent intratumor clonotypes in responder patients were also significantly present in the matched blood and normal lung tissue, while this association was not significant in non-responder patients. This finding indicating that more migratory T cell clones correlated with antitumor response. Finally, the authors found a significant association between the increased number of tumor-infiltrating lymphocytes and the dynamic changes in frequency of peripheral clonotypes shared with the tumor, a findings suggestive of an active compartmental exchange of intratumor clonotypes induced by neoadjuvant PD-1 blockade. Notably, peripheral T cell clonotypic expansion between weeks 2-4 after neoadjuvant anti-PD1 treatment initiation correlated with greater intratumoral clonotype accumulation for patients with response. On the contrary, non-responder tumors did not successfully traffic top 1% intratumor clonotypes to the tumor bed, possibly due to an intrinsically more exhausted, less migratory T cell repertoire. This phenomenon supports the idea that the formation of T-cell clones committed against the tumor can expand to other tissues in order to fight micrometastasis, thereby providing a justification for the use of immune checkpoint inhibitors prior to surgery [75,98].

### 6.3. Squamous Cell Carcinoma

A biological question that is particularly suitable to be explained by immune repertoire analysis is whether the T cell response to checkpoint blockade relies on reinvigoration of pre-existing tumor-infiltrating lymphocytes or on recruitment of novel T cells. This question has been elegantly addressed by Yost K.E. et al. [99], through paired single-cell RNA and TCR sequencing on cells from site-matched tumors from patients with basal or squamous cell carcinoma before and after anti-PD-1 therapy. This methodological approach allowed the author to observe that anti-PD-1 treatment resulted in an increased frequency of activated and exhausted CD8+ T cells in tumors, supporting the notion that PD-1 blockade primarily affects CD8+ T cells. Clonality analysis revealed that exhausted CD8+ T cells had the highest levels of clonality compared with all other CD8+ T cells (naive, memory, effector memory, activated, chronically activated/exhausted, and intermediate exhausted/activated). Moreover, clonally expanded T cells shared a common exhausted phenotype that correlated with antigen specificity, that maintained stable in response to anti-PD1. Most of these exhausted clones detected after anti-PD1 treatment were derived from clones that had not been detected before treatment. Of note, one-third of the novel exhausted clonotypes were detected in peripheral blood, indicating a possible approach to monitor tumor-specific T cell responses to anti-PD1 treatment [99].

### 6.4. Prostate and Urothelial Cancers

The T-cell clonality (1—Pielou’s eveness) in pretreatment blood samples did not correlate with clinical benefit or toxicity outcomes upon CTL4A treatment of metastatic prostate cancer [23]. However, an increase of clonality, underlying an expansion of a mono- or oligo-clonal population, has been shown to precede immune-related adverse events.

In metastatic urothelial cancer, TCR clonality below the median in the peripheral blood prior to treatment, and expansion of tumor-associated TCR in the periphery 3 weeks after initiating treatment, are all associated with clinical benefit [74]. The authors speculated that low clonality of TCR in the blood prior to treatment may increase the likelihood that a patient will host one or more clones capable of recognizing the tumor. The expansion of peripheral blood tumor-associated TCRs highlights the continuity of the blood compartments and tumor tissue, and suggests that PD-L1 block activity may involve circulating T cells more than previously thought [74].

## 7. Conclusions

The extraordinary success of anticancer immunotherapies targeting the immune checkpoint molecules, CTLA-4, PD-1 and PD-L1 has strengthened awareness of the essential role of the immune system in eradicating tumors. The ability to predict whether a patient will respond or become resistant to immunotherapy is increasingly benefiting from biomarkers that reflect the state of the T cell repertoire. Key factors that may contribute to a better understanding of the impact of immunotherapies on the patient adaptive immune system appears to be TCR diversity and clonality. At present, the major weakness of these biomarkers is the lack of rigorous procedures for their calculation, mainly due to the different methodologies used for the analysis of the T cell repertoire, further emphasizing the need for their optimization and validation. As many new combination therapies are developing, TCR repertoire analysis should be studied, alone or in combination with other immune parameters, as source of biomarkers of response, and to further elucidate the mechanisms of successful treatment.

## Figures and Tables

**Table 1:** Non-exhaustive list of companies providing immune repertoire products and services.

Company	Kit/Service	Starting Material	Library Preparation	Chains	Sequencing Platform
ThermoFisher Sci.	Oncomine TCR Beta	DNA/RNA	Multiplex PCR primers FR1-C	β	Iontorrent
Takara	SMARTer Human TCRα/β Profiling Kit	RNA	5′ RACE	α/β	ILLUMINA
Adaptive Biotechnologies	ImmunoSEQ	DNA	Multiplex-PCR primers V-J	α/β/δ/γ	ILLUMINA
BGI (Copenhagen N, Denmark)	IR-SEQ	RNA	Multiplex PCR or 5′ RACE	α/β	ILLUMINA
CD Genomics (New York, USA)	Immune Repertoire Sequencing	DNARNA	Multiplex PCR or 5′ RACE	α/β	ILLUMINA
iRepertoire, Inc. (Huntsville, USA)		DNARNA	Multiplex PCR primers V-J or V-C	α/β/δ/γ	ILLUMINA

**Table 2:** Exemplary bioinformatics tools for TCR repertoire analysis.

Tools	Data Format	PCR/Sequencing Error Correction	Accessibility ^1^	Reference
IMGT/HighV-Quest	FASTA	NO	Web	[55]
MiXCR	FASTA/FASTQ	YES	SA	[56]
MiTCR	FASTQ	YES	SA	[57]
Vidjil	FASTA/FASTQ	YES	Web/SA	[58]
IMSEQ	FASTA/FASTQ	YES	SA	[59]
RTCR	FASTQ	YES	SA	[60]
TRIg	FASTA	NO	SA	[61]

^1^ Web-based or standalone (SA) version that can be implemented within a computer.

**Table 3:** TCR repertoire metrics used as biomarkers in major checkpoint blockade immunotherapy studies.

Reference	Disease	CBI	TCR Repertoire Metrics
Robert, L. et al. [71]	melanoma	CTLA4 (tremelimumab)	richness, Shannon diversity index, Pielou’s evenness index
Cha, et al. [72]	melanoma, prostate	CTLA4 (ipilimumab)	top 25th percentile clonotypes, Morisita’s distance
Tumeh, P.C. et al. [73]	melanoma	PD-1 (pembrolizumab)	Shannon entropy, 1-normalized entropy
Snyder, A. et al. [74]	urothelial	PD-L1 (atezolizumab)	Shannon entropy, 1-normalized entropy
Forde, P.M. et al. [75]	NSCLC^1^	PD-1 (nivolumab)	1-normalized entropy
Yusko, E. et al. [76]	melanoma	PD-1/CTLA4 (nivo/ipilimumab)	1-normalized entropy
Postow, M.A. et al. [77]	melanoma	CTLA4 (ipilimumab)	richness, evenness index
Hogan, S.A. et al. [78]	melanoma	PD-1/CTLA4	diversity evenness score (DE50)
Hopkins, A. et al. [79]	pancreatic ductal adenocarcinoma	CTLA4 (ipilimumab)	Morisita’s distance, (1-normalized entropy)
Roh, W. et al. [80]	melanoma	PD-1/CTLA4 (nivo/ipilimumab)	Shannon entropy, TCR clonality
Subudhi, S.H et al. [81]	prostate	CTLA4 (ipilimumab)	Shannon entropy, 1-normalized entropy
Han, J. et al. [82]	NSCLS	PD-1/PD-L1	Shannon entropy, 1-normalized entropy
Khunger, A. et al. [83]	melanoma	CTLA (tremelimumab)	1- Pielou’s Evenness, Morisita’s distance
Looney, T.J. et al. [84]	Clear cells, melanoma, prostate	CTLA	Shannon entropy, TCR Convergence

^1^ NSCLC, non–small-cell lung cancer; SSC, squamous cell carcinoma.

## Abbreviations

APC	Antigen presenting cell
CBI	checkpoints blockade immunotherapy
CDR3	complementarity-determining region 3
HTS	high-throughput sequencing
MHC	major histocompatibility complex
NSCLC	non–small-cell lung cancer
PCR	polymerase chain reaction
RACE	rapid amplification of cDNA ends
SSC	squamous cell carcinoma
TCR	T cell receptor
UMIs	unique molecular identifiers
VDJ	variable (V), diversity (D), and joining (J) gene segments
